# Reduced wear in vitamin E-infused highly cross-linked polyethylene cups: 5-year results of a randomized controlled trial

**DOI:** 10.1080/17453674.2020.1852785

**Published:** 2020-12-02

**Authors:** Goulven Rochcongar, Matthieu Remazeilles, Emeline Bourroux, Julien Dunet, Valentin Chapus, Matthieu Feron, César Praz, Geoffrey Buia, Christophe Hulet

**Affiliations:** Caen Normandy University Hospital Centre, Department of Orthopaedic and Trauma Surgery, 14000, Caen, France

## Abstract

Background and purpose — Vitamin E-infused polyethylene is a relatively new material in joint arthroplasty; there are no long-term reports, and only few mid-term results. Using radiostereometric analysis (RSA), we primarily determined whether vitamin E-infused highly cross-linked polyethylene (HXLPE/VitE) acetabular cups show less wear than ultra-high molecular weight polyethylene (UHMWPE) acetabular cups at 5 years after total hip arthroplasty (THA). We also assessed whether wear rates correlate with increasing cup inclination angles or cup sizes.

Patients and methods — This is a 5-year follow-up of our previously reported randomized controlled trial of 62 patients with 3 years’ follow-up, who received THA with either an HXLPE/VitE or a UHMWPE acetabular cup. At 5 years, 40 patients were analyzed (22 in the HXLPE/VitE and 18 in the UHMWPE group).

Results — HXLPE/VitE cups continued to show less cumulative femoral head penetration than UHMWPE cups (HXLPE/VitE: 0.24 mm, UHMWPE: 0.45 mm; p < 0.001). Distribution of wear was also more even with HXLPE/VitE cups than with UHMWPE cups (p = 0.002). Moreover, the difference in PE wear between 1 and 5 years in both groups showed no statistically significant correlation with increasing cup inclination angles or cup sizes. Finally, no osteolysis and implant loosening occurred, and no revision surgeries were required.

Interpretation — Wear rates continue to be lower in HXLPE/VitE cups than in UHMWPE cups at 5 years of follow-up without correlation with increasing cup inclination angles or cup sizes. Finally, HXLPE/VitE cups may have the potential to prevent osteolysis and implant loosening.

Wear of the polyethylene (PE) component of total hip arthroplasties (THA) may result in osteolysis (Callary et al. 2015). Therefore, attempts such as cross-linking using irradiation and addition of vitamin E have been made to improve the wear properties of PE (Galea et al. 2019). Vitamin-E infused highly cross-linked polyethylene (HXLPE/VitE) has been developed to reduce the number of free radicals without compromising the mechanical properties. There are 2 methods of incorporating vitamin E into PE. The 1st is to blend vitamin E with PE powder before consolidation and. Once consolidated, the blend can be irradiated for sterilization or cross-linking. The 2nd is diffusion of vitamin E into the PE after radiation cross-linking: after PE is irradiated for cross-linking, it is diffused with vitamin E, then machined into its final form and gamma sterilized (Oral et al. 2005). Gamma irradiation causes crosslinking of UHMWPE, which changes its property from the original. However, it causes reduction in tensile strength and elongation of UHMWPE, and leads to long-lived free radicals that react with oxygen (Oral et al. 2007).

As HXLPE/VitE is a relatively new material in orthopedic surgery, studies on its wear properties with longer follow-up periods are still limited (Nebergall et al. 2016, 2017, Shareghi et al. 2017, Galea et al. 2019). Our initial 3-year data showed less wear with HXLPE/VitE, which may prevent osteolysis, implant loosening, and eventually revision surgery (Rochcongar et al. 2018). We have now investigated clinical and radiographic outcomes of our previously reported patient cohort at 5-year follow-up. The primary objective is to know whether HXLPE/VitE acetabular cups continue to show less PE wear than ultra-high molecular weight polyethylene (UHMWPE) acetabular cups at 5 years. The secondary objective is to evaluate the correlation between PE wear rates with cup inclination angles or cup sizes, in addition to reporting clinical outcomes. 

## Patients and methods

### Trial design

This single-center, randomized controlled trial (RCT) was undertaken as a stratified, parallel-group RCT.

### Patients

Inclusion and exclusion criteria were the same as described previously (Rochcongar et al. 2018). The study was performed at Caen University Hospital, a major referral hospital.

### Randomization and blinding

Randomization and blinding were carried out as described previously (Rochcongar et al. 2018).

### Interventions

On the acetabular side, patients received either an HXLPE/VitE cup (RM Pressfit vitamys, Mathys Ltd, Bettlach, Switzerland) or a UHMWPE cup (RM Pressfit, Mathys Ltd, Bett­lach, Switzerland).

All procedures were performed by attending surgeons and fellows under supervision.

### Outcomes

The primary and secondary outcomes were set a priori and measured as described previously (Rochcongar et al. 2018). As the primary outcome, the femoral head penetration using model-based RSA with the patient standing on both legs (Callary et al. 2015) was measured. To obtain suitable images of the hip, ceiling-mounted and mobile radiographic tubes were used simultaneously, with a calibration cage behind the patient. RSA measurements prior to the study were validated by 3 blinded investigators who performed the measurements of specially manufactured liners with different concentric wear rates. The precision was to be 0.072 mm and accuracy to be 0.034 mm, which is similar to the values reported in previous studies (Pineau et al. 2010). The RSA exam was performed 7 days after surgery (baseline) and then again at 6 months and at 1, 2, 3, and 5 years later.

RSA images were analyzed using Medis Specials medical imaging software (Medis, Leiden, the Netherlands). All images were processed using contour detection software (Model-Based RSA [MBRSA], version 3.2; Medis) as described previously (Garling et al. 2005). The 3D contour of each implant was projected on each view. Wear was calculated by taking the 12-month mark as the baseline for the measurements, and all changes in relative distance between the head and cup were assumed to be due to creep and possible wear. The initial distance between the center of the cup and the center of the head was defined as the minimal head penetration into the liner. 3D femoral head penetration was calculated as the vectorial sum of medial (x), proximal (y), and anterior translation (z) (Callary et al. 2013). The results were expressed as the global femoral head penetration rather than separating the 3 vectors as described by Önsten et al. (1998), with radiographs made with the patient in the standing position. Linear motion was our main interest and volumetric wear was not considered.

As secondary outcomes, clinical scores were assessed preoperatively as well as postoperatively whenever RSA was performed; they included the Harris Hip Score (HHS) and Merle d’Aubigné and Postel (MAP) score. Radiographically, the mean cup inclination angle on the anteroposterior pelvic radiograph was measured postoperatively and during the follow-up period. PE wear was measured at 1 and 5 years in correlation with the cup inclination angles (ranging from 20° to 60°) and cup sizes (ranging from 48 to 62 mm). Postoperative complications were reported.

### Statistics

The investigation was constructed as a superiority study. Statistical analysis was performed as described previously (Rochcongar et al. 2018).

All patients were included in the analysis, regardless of the actual surgery performed, according to the intention-to-treat principle. We used the Mann–Whitney U-test to compare the endpoints and measured the Spearman coefficient to detect a correlation between PE wear and cup inclination angles or cup sizes. For participants who were withdrawn from the study before completion, data from the last observation was carried forward (imputed). All statistical analyses were performed with the statistical software StatView (SAS Institute, Cary, NC, USA). P-values of less than 0.05 were considered as significant.

### Ethics, registration, funding, and potential conflicts of interest

All enrolled patients gave informed consent to participate in the study. This study was approved by the North West French regional ethics committee (Comité de Protection des Personnes Nord-Ouest III; study number: 2009-A00948-49). An independent administrative body (Commission Nationale de l’Informatique et des Libertés) ensured data protection. This study is in compliance with the latest recommendations in the Helsinki Declaration and Public Health Act No. 2004-806. It was registered at clinicaltrials.gov (identifier: NCT02524587). Caen University Hospital received funding from Mathys Ltd Bettlach to finance part of the study costs, which included RSA analysis and data collection. Mathys Ltd Bettlach had no role in the design or execution of the study, the analysis or interpretation of the data, or the decision to submit results. CH was paid speaker for Mathys. No other authors declare any conflict of interest in connection with the submitted article. 

## Results

### Patients

**Figure 1.**
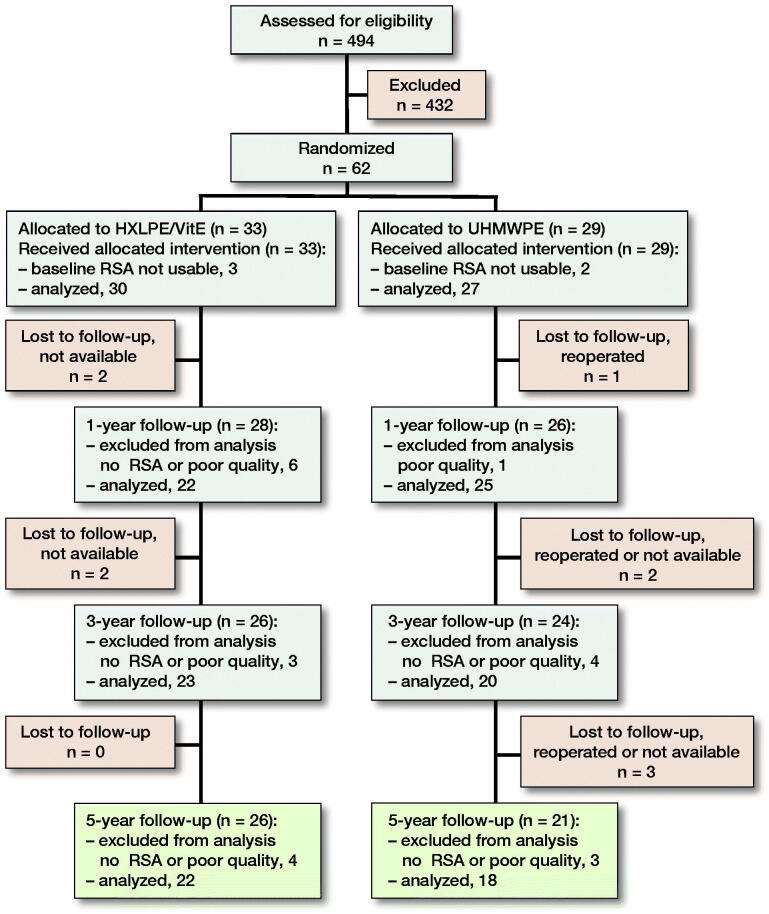
Study enrollment. The reasons for the losses to follow-up—if known—are indicated in the corresponding boxes. HXLPE/VitE: Vitamin E-infused highly cross-linked polyethylene, RSA: radiostereometric analysis, UHMWPE: ultra-high molecular weight polyethylene.

494 patients were enrolled in the study between January 2010 and November 2011. After exclusion of 432 patients, 62 of them were randomized into the 2 groups, with 29 patients in the UHMWPE group and 33 patients in the HXLPE/VitE group (Figure 1). Finally, 40 patients were analyzed at 5 years, which included 22 patients in the HXLPE/VitE group and 18 patients in the UHMWPE group.

Most patients underwent THA for osteoarthritis. At baseline, the 2 groups were similar with respect to patient age, sex, BMI, or any of the clinical scores (Table 1).

**Table 1.** Baseline demographics for the 2 patient groups

	HXLPE/VitE	UHMWPE
Factor	(n = 33)	(n = 29)
Mean age (SD)	61 (6.5)	61 (7.8)
Female sex	16	17
BMI (SD)	27 (4.1)	27 (3.7)
Indication		
Primary or secondary osteoarthritis	31	26
Osteonecrosis	2	3
Surgical approach		
Anterolateral approach		
(modified Harding)	18	14
Posterior approach (Moore)	13	14
Trochanter osteotomy	2	1
Preoperative clinical scores (SD)		
Harris Hip Score	52 (11)	53 (12)
Merle d’Aubignй and Postel score	13 (1.7)	12 (3)

HXLPE/VitE: Vitamin E-infused highly cross-linked polyethylene. UHMWPE: ultra-high molecular weight polyethylene.

### Primary outcome: femoral head penetration

**Figure 2.**
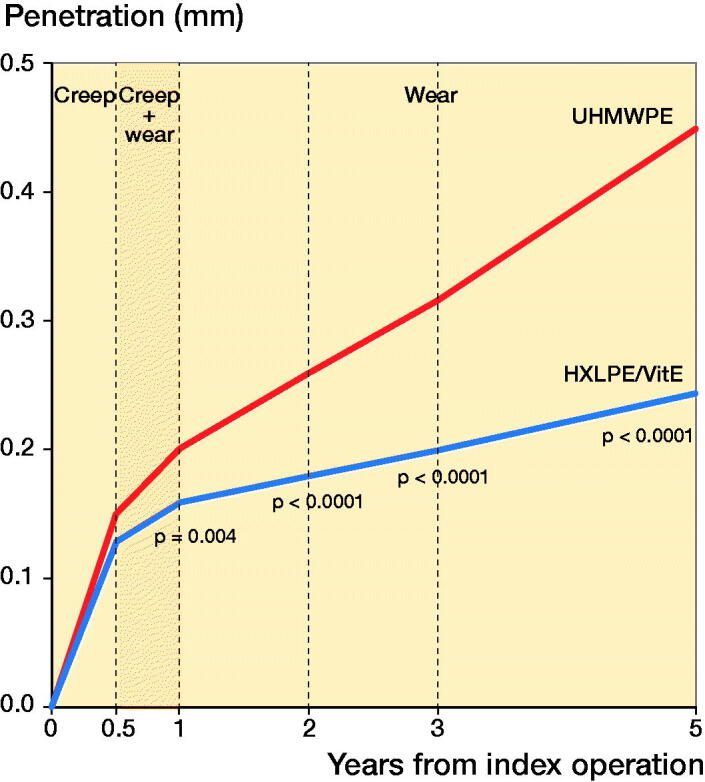
Creep and wear behavior of UHMWPE (n = 18, red line) and HXLPE/VitE (n = 22, blue line) over the first 5 years after implantation. HXLPE/VitE: Vitamin E-infused highly cross-linked polyethylene, UHMWPE: ultra-high molecular weight polyethylene.

5 years after surgery, the cumulative femoral head penetration was significantly lower in the HXLPE/VitE group than in the UHMWPE group (p < 0.001) (Table 2, Figure 2). From 1 to 5 years after surgery, the mean femoral head penetration increased 0.08 mm in HXLPE/VitE cups, while it increased 0.2 mm in UHMWPE cups. The wear rate averaged 0.02 mm/year in the HXLPE/VitE group compared with 0.06 mm/year in the UHMWPE group. The estimated steady-state rate of wear was thus approximately 66% lower in the HXLPE/VitE group than in the UHMWPE group (p < 0.001).

**Table 2.** Cumulative femoral head penetration. Values are mean (SD)mm

	Years after total hip arthroplasty		
Group	1	3	5
HXLPE/VitE	0.16 (0.03)	0.20 (0.03)	0.24 (0.04)
UHMWPE)	0.20 (0.05)	0.32 (0.07)	0.45 (0.13)
P-value **^a^**	0.004	< 0.001	< 0.001

HXLPE/VitE: Vitamin E-infused highly cross-linked polyethylene. UHMWPE: ultra-high molecular weight polyethylene.

**^a^** Mann–Whitney U-test.

HXLPE/VitE cups also showed a more even distribution of wear than UHMWPE cups (p = 0.002). At 5 years after surgery, the coefficient of variation, calculated by the standard deviation divided by the mean, was 28 in HXLPE/VitE cups and 18 in UHMWPE cups, which translated to an approximately 50% lower variation of wear in HXLPE/VitE cups than in UHMWPE cups.

### Secondary outcomes: clinical results and complications

From preoperative values to 5 years after surgery, both the HHS and the MAP score improved in both groups (p < 0.001). 5 years after surgery, none of the mean clinical scores differed statistically significantly between the HXLPE/VitE group and the UHMWPE group (HHS 97 [SD 8] versus 99 [SD 3], p = 0.4; MAP score 18 [SD 1] versus 18 [SD 0.4], p = 0.3).

At 5 years, the mean cup inclination angle was similar in both groups (HXLPE/VitE 48° [SD 7°], UHMWPE 46° [SD 6°]; p = 0.3) and remained stable over the entire follow-up period (HXLPE/VitE range, 48° to 49°, UHMWPE range, 46° to 48°). Moreover, the difference in PE wear between 1 and 5 years in both groups showed no significant correlation with increasing cup inclination angles (HXLPE/VitE r = 0.2, p = 0.5; UHMWPE r = 0.1, p = 0.8) or cup sizes (HXLPE/VitE r = 0.02, p = 1.0; UHMWPE r = –0.1, p = 0.7).

No complications occurred during the first 5 years of follow-up. 

## Discussion

We confirmed continued good clinical and radiographic outcomes with HXLPE/VitE cups at 5 years after THA. From 1 to 5 years, femoral head penetration increased in both groups; however, the wear rate was 66% lower in HXLPE/VitE cups than in UHMWPE cups. This means that the difference in wear rate between the two groups remained highly significant at 5 years (p < 0.001). Based on the linearity of the curves between 1 and 5 years (Figure 2), one would expect the same trend to continue over time, further increasing the difference between the two groups.

The mean femoral head penetration of HXLPE/VitE cups, from 1 to 3 years after surgery, increased 0.04 mm compared with 0.12 mm in UHMWPE cups (Rochcongar et al. 2018). Overall, the wear rate averaged 0.020 mm/year in the HXLPE/VitE group and 0.058 mm/year in the UHMWPE group. The estimated steady-state rate of wear was thus 65% lower in the HXLPE/VitE group than in the UHMWPE group (p < 0.001). The same trend continued at 5 years.

PE wear is associated with the onset of osteolysis after THA (Dumbleton et al. 2002, Bitar and Parvizi 2015). According to Elke and Rieker (2018), a wear rate of 0.1 mm/year for any femoral head size correlates with an osteolysis-free survival of less than 20 years. Dowd et al. (2000) found that osteolysis did not develop in patients after THA using non-cross-linked PE devices with a wear rate of < 0.1 mm/year, while Dumbleton et al. (2002) suggested that osteolysis was almost absent with a wear rate of 0.05 mm/year. Our wear rate of 0.02 and the more even distribution of wear in the HXLPE/VitE group is therefore promising for clinical outcomes, such as osteolysis over longer follow-up periods.

Clinical evidence suggests that cup inclination angles and cup sizes are correlated with PE wear, with inclination angles of ≥ 45° and cup sizes of ≥ 58 mm leading to increased PE wear with time (Bono et al. 1994, Little et al. 2009, Tian et al. 2017 and 2018). Although the ideal cup inclination angle has not yet been established, it is accepted that suboptimal acetabular positioning can lead to accelerated wear (Little et al. 2009, Tian et al. 2017, 2018). In our study, both HXLPE/VitE and UHMWPE cups showed no significant correlation of the PE wear rate with increasing cup inclination angles. A biomechanical study comparing the wear rates of HXLPE/VitE and UHMWPE cups found that the wear rates of HXLPE/VitE cups remained similar at standard (45°) and at the highest possible inclination angle (80°), suggesting that HXLPE/VitE cups accommodate implant malorientation better than UHMWPE cups (Halma et al. 2014). Teeter et al. (2018) also found no correlation between cup inclination angle and PE wear of HXLPE acetabular cups: PE wear of cups within the target inclination angle (40°) and outside of it (47.5°) was the same (0.05 mm/year), suggesting that HXLPE is a forgiving bearing material in terms of wear. Additionally, in our study the PE wear rate showed no statistically significant correlation with increasing cup sizes. In the monoblock implants used, the PE thickness depends on the cup size, which means that PE wear was not correlated with the PE thickness.

So far, studies have reported outcomes of vitamin E-stabilized PE liners (Berend et al. 2015, Lindalen et al. 2015, Salemyr et al. 2015, Shareghi et al. 2015, Sillesen et al. 2015, Nebergall et al. 2016, Sillesen et al. 2016, Nebergall et al. 2017, Scemama et al. 2017, Shareghi et al. 2017, Wyatt et al. 2017, Busch et al. 2019, Galea et al. 2019).

There are the limitations of our study. We were unable to isolate the effect of vitamin-E stabilization from irradiation-induced cross-linking, because the two cups were built from differently cross-linked PEs. It is known that cross-linking has a beneficial effect on wear, while vitamin E is expected to hinder aging (Lindalen et al. 2015, Salemyr et al. 2015, Nebergall et al. 2016, 2017, Scemama et al. 2017, Galea et al. 2019). However, we assessed none of these effects. Therefore, further clinical trials with identically cross-linked PEs and longer follow-up periods are required to estimate the true benefits of vitamin-E infusion. Overall, 10 patients were lost to follow-up, which can be expected in any long-term follow-up study. Finally, the small sample size may have limited us in detecting highly significant correlations of PE wear and varying cup inclination angles or cup sizes.

In conclusion, the wear rate continues to be lower in HXLPE/VitE cups than in UHMWPE cups at 5 years of follow-up. The steady-state wear rate for HXLPE/VitE cups was more than 5 times below the critical value reported as leading to osteolysis. Additionally, wear rates had no correlation with increasing cup inclination angles or cup sizes. Therefore, this study confirms that HXLPE/VitE cups have the potential to prevent osteolysis, implant loosening, and eventually revision surgery in the future.
